# Identity Reconstruction Through Leisure Activities in Parkinson's Disease: A Three-Case Qualitative Report

**DOI:** 10.7759/cureus.115332

**Published:** 2026-08-27

**Authors:** Mitsushi Sekimoto, Reina Motoori, Mitsuhiro Nito, Hiromi Fujii

**Affiliations:** 1 Occupational Therapy, Graduate School of Health Sciences, Yamagata Prefectural University of Health Sciences, Yamagata, JPN; 2 Community Rehabilitation Division, Linie L, Inc., Osaka, JPN

**Keywords:** depressive symptoms, family support, leisure participation, parkinson's disease, value reconstruction

## Abstract

Depression, a major non-motor manifestation of Parkinson's disease (PD), substantially reduces patients' quality of life. Although leisure activities may protect against the progression of psychiatric symptoms, clinical observations indicate that some patients discontinue such activities and subsequently develop severe depressive symptoms despite preserved physical function. This three-case qualitative report aims to describe and qualitatively analyze the common characteristics, divergent patterns of leisure engagement, and psychological adaptation process in three home-care patients with Hoehn and Yahr Stage III PD.

Three home-care patients with PD at Hoehn and Yahr Stage III were included. Qualitative analysis was performed using the Steps for Coding and Theorization method. Physical function was evaluated using the Movement Disorder Society-sponsored revision of the Unified Parkinson's Disease Rating Scale (MDS-UPDRS) Part III, whereas depressive symptoms were assessed using the Japanese version of the 15-item Geriatric Depression Scale (GDS-15-J).

Case 3, who demonstrated the most favorable motor function score (MDS-UPDRS Part III: 10), discontinued leisure activities because of excessive risk-avoidance attitudes among family members and subsequently developed depressive symptoms (GDS-15-J: score 10). In contrast, Cases 1 and 2, despite exhibiting more severe motor impairment (29 and 17, respectively), continued their activities by redefining these activities as "practice for maintaining function" or "preservation of social roles." Supported by appropriate family involvement, these patients maintained participation and exhibited no depressive symptoms (GDS-15-J: 1 and 2, respectively).

The findings suggest that preservation of mental health in home-care patients with PD is influenced more strongly by subjective reinterpretation of activity value and risk-inclusive family support than by residual physical capacity.

In conclusion, this three-case qualitative report describes how home-care patients with Stage III PD adapt to progressive functional decline through leisure activities. Our findings suggest that sustained engagement in meaningful occupations is supported not only by individual cognitive restructuring but also by collaborative family understanding. For multidisciplinary healthcare teams providing home-based care, recognizing the subjective value patients attach to leisure activities may help facilitate psychological well-being and prevent depressive symptoms.

## Introduction

Parkinson's disease (PD) is a progressive neurodegenerative disorder characterized by motor symptoms such as tremor and bradykinesia, along with a high prevalence of non-motor symptoms, including depressive symptoms, anxiety, and apathy [1]. These non-motor symptoms substantially impair activities of daily living and quality of life (QOL) among individuals with PD receiving home-based care [2]. Consequently, preserving psychological well-being, conceptualized in this study as a multidimensional state encompassing subjective life satisfaction, emotional stability, and a sense of purpose through meaningful occupations, beyond the mere absence of depressive symptoms (as screened by the Japanese version of the 15-item Geriatric Depression Scale (GDS-15-J < 5)), remains a major challenge for these patients in maintaining participation in community life.

Leisure activities have been recognized as important contributors to the maintenance of physical and psychological function and to the enhancement of QOL [3,4]. In our previous study examining the association between the duration of leisure activity engagement and non-motor symptoms in patients with home-care PD, longer participation in leisure activities was negatively correlated with anxiety and depressive symptoms [5]. These findings suggest that sustained engagement in meaningful leisure activities may play a significant role in supporting the mental health of individuals with PD.

However, considerable differences are observed in clinical practice. Some patients receiving home-based care continue to participate in leisure activities even at the same disease severity level (Hoehn and Yahr Stage III) [6]. In contrast, others discontinue such activities due to disease progression, environmental changes, or insufficient understanding and support from those around them. While existing literature has demonstrated the general benefits of activity engagement in PD, how home-care patients adapt to progressive motor and non-motor challenges in their daily leisure activities remains insufficiently understood. We hypothesized that an individual's capacity to reconstruct the subjective meaning of leisure activities-shifting focus from physical performance to emotional, cognitive, and social value-might represent a key factor in psychological adaptation and well-being. Most previous studies have focused primarily on activity frequency or on associations with physical function. In contrast, relatively few have examined in detail how environmental factors, such as family understanding and patients’ subjective attributions of meaning, influence decisions to continue or discontinue activities [7,8]. While previous clinical studies have primarily evaluated the functional and motor outcomes of activity engagement in PD [1,2,5], the psychosocial processes of subjective meaning-making and value reconstruction remain less explored [4]. Emerging occupational and qualitative frameworks suggest that adapting the personal significance of activities may help individuals navigate chronic identity disruption [7,9]; however, how home-care patients specifically negotiate these processes amidst progressive motor and non-motor symptoms requires further descriptive observation.

Therefore, this report presents three cases of home-care patients with PD who experienced different trajectories regarding the continuation or discontinuation of leisure activities. To address this knowledge gap, this three-case qualitative report aimed to describe and qualitatively analyze the common characteristics, divergent patterns of leisure engagement, and psychological adaptation processes in three home-care patients with Hoehn and Yahr Stage III PD, using the Steps for Coding and Theorization (SCAT) method [10]. Potential clinical implications and theoretical interpretations are addressed separately in the Discussion section. Through these case reports, we discuss the importance of respecting individual values, identifying psychological changes associated with difficulties in maintaining activities at an early stage, and implementing practical evaluation and support strategies for occupational therapy interventions.

## Case presentation

Selection of participants and ethical considerations

A total of 35 home-care patients with PD, recruited from a home-visit nursing agency and a PD-specialized day-care facility, were initially screened. From this screened pool, three cases were purposively selected by two experienced occupational therapists using information-rich sampling to capture contrasting patterns of leisure activity engagement among patients with Stage III PD (Table 1): one who initiated a new leisure activity (Case 1), one who continued their existing leisure activity (Case 2), and one who discontinued their leisure activity due to functional decline (Case 3). To minimize selection and confirmation bias, selection criteria were restricted to pre-established, objective behavioral patterns of leisure engagement (initiating, continuing, or discontinuing) observed in routine clinical practice prior to qualitative coding. Case selection was conducted collaboratively by two experienced occupational therapists independently of the subsequent SCAT thematic analysis to prevent selecting cases based on theoretical expectations.

**Table 1:** Clinical and behavioral characteristics of the three cases. BI: Barthel Index; MDS-UPDRS: Movement Disorder Society-sponsored revision of the Unified Parkinson's Disease Rating Scale; GDS-15-J: 15-item Geriatric Depression Scale-Japanese version; MoCA-J: Montreal Cognitive Assessment-Japanese version. Note: BI scores measure functional independence in basic activities of daily living (ranging from 0 to 100, where higher scores indicate greater independence). Higher scores on the MDS-UPDRS indicate greater motor severity. On the GDS-15-J (maximum score: 15), scores of 5 or higher suggest a risk of depression. Regarding the MoCA-J (maximum score: 30), scores of 26 or higher are considered cognitively unimpaired, while scores between 18 and 25 indicate mild cognitive impairment (MCI). Case 1 (MoCA-J score: 23) met inclusion criteria within the MCI range without clinical dementia. Preserved communicative fluency and reflective capacity required for qualitative interviewing were clinically confirmed through observational interaction during routine occupational therapy sessions by experienced therapists, rather than inferred solely from the MoCA-J score.

Parameter	Case 1	Case 2	Case 3
Age/sex	Late 60s/Female	Early 70s/Female	Early 70s/Female
Disease duration (years)	10	8	12
Hoehn and Yahr stage	Ⅲ	Ⅲ	Ⅲ
BI	80	90	85
MDS-UPDRS Part I	6	5	10
MDS-UPDRS Part III	29	17	10
GDS-15-J	1	2	10
MoCA-J	23	27	29
Leisure activities	Coloring, Saxophone, Table tennis	Dance	Handicraft (discontinued)

This study was approved by the Ethics Committee of Linie R, Inc. (Approval No. 2091) and was conducted in accordance with the principles of the Declaration of Helsinki. Written informed consent was obtained from all participants prior to study participation, and all personally identifiable information was anonymized to ensure confidentiality.

Assessments and analysis

This study employed a cross-sectional qualitative case series design. Upon enrollment and obtaining written informed consent, all clinical assessments and qualitative interviews were completed within a single designated evaluation session on the same day.

Motor and non-motor symptoms were evaluated using Parts I and III of the Movement Disorder Society-sponsored revision of the Unified Parkinson's Disease Rating Scale (MDS-UPDRS) [11]. All motor function evaluations using the MDS-UPDRS Part III were systematically performed during the medication "ON" state, approximately one to two hours following the administration of anti-Parkinsonian medication. Depressive symptoms and cognitive function were screened using the Japanese versions of the 15-item Geriatric Depression Scale (GDS-15-J) [12] and the Montreal Cognitive Assessment (MoCA-J) [13], respectively. Baseline functional independence in basic activities of daily living was evaluated using the Barthel Index.

Immediately following clinical scoring, a single face-to-face semi-structured qualitative interview (40-60 minutes) was conducted individually with each participant by one licensed occupational therapist. The interview focused on the patient's leisure activity engagement, subjective experiences, coping processes, and family relationships over the preceding one month, using a questionnaire uniquely developed by the authors (Table 2). With participant consent, all interviews were audio-recorded and transcribed verbatim in Japanese for SCAT coding.

Structural analysis was performed using the SCAT method. This qualitative analytical approach consists of four stages: (1) segmentation and coding, (2) incorporation of external concepts, (3) contextualization, and (4) theorization. The method is particularly useful for describing and theorizing psychosocial processes based on a limited number of cases. Participant characteristics are presented in Table 1. Notably, Case 1 demonstrated the most severe motor impairment (MDS-UPDRS Part III score: 29) but exhibited the most favorable psychological status (GDS-15-J score: 1). The psychosocial processes underlying this discrepancy between physical severity and psychological well-being are described below.

**Table 2:** Semi-structured interview questionnaire regarding leisure activities. This semi-structured interview questionnaire was originally developed by the authors to deeply explore the participants' lived experiences and the psychological or social factors influencing their occupational engagement. Individual interviews were conducted in a private, quiet room and lasted approximately 40 to 60 minutes per session.

No.	Interview questions
Q1	Have you engaged in any leisure activities within the past month? (Yes/No)
Q2-1	If yes: Please describe the specific details and content of the leisure activities you engaged in during the past month.
Q2-2	If no: Please describe the specific details and content of the leisure activities you used to engage in previously.
Q3	What factors or support do you believe enabled you to continue your leisure activities? (Alternatively, if activities were discontinued, what kind of support do you think would have helped you continue?)
Q4	Since the onset of Parkinson's disease, what changes have you experienced in your participation or approach to these leisure activities?
Q5	What kind of value or meaning do these leisure activities hold for you?

Case 1: Psychological adaptation model through meaning-making

Case 1 was a woman in her late 60s who had been living with PD for 10 years, presenting with preserved cognitive function (MoCA-J: 23/30) and no depressive symptoms (GDS-15-J: 1/15). Following her diagnosis, she initiated several new leisure activities, including coloring, playing the saxophone, and table tennis. Initially, she experienced internal distress due to a strong preoccupation with the quality and perfection of her performance. Over time, however, she shifted her evaluation criteria toward functional maintenance and personal meaning. She reframed coloring as foundational practice for handwriting, playing the saxophone as muscle training for abdominal breathing and oral function, and table tennis as balance practice that also expanded her social circle. Furthermore, using home-visit helper services enabled her to develop a broader support network beyond her family. Learning that her care worker's family member also had PD, she felt that interacting with her provided an opportunity for the worker to learn about PD support, giving her a sense of altruistic purpose. She also received substantial emotional support from her husband, who listened attentively to her feelings and learned the saxophone alongside her.

Reflecting on these experiences, she stated: "Rather than focusing on completing coloring neatly or playing saxophone tunes perfectly, I came to realize that treating them as foundational exercises for writing or muscle training for abdominal breathing was far more meaningful for me," and "My husband always listens quietly and thinks through things with me, which is why I can keep going without giving up."

Steps for Coding and Theorization (SCAT) Analysis Results

This case illustrates a "psychosocial adaptation model" for maintaining quality of life. SCAT analysis revealed three major categories (Table 3):

1. Cognitive Restructuring and Re-meaning: Shifting subjective criteria from external achievement and perfection toward functional maintenance and personal meaning.

2. Self-Determined Engagement: Expanding reliance beyond family to formal care providers (helpers) and transforming her identity from a passive recipient into a supportive contributor who helps others understand PD.

3. Spousal Buffer Effect: The spouse acting as an empathetic listener and collaborative partner, which was derived directly from verbatim quotes regarding his quiet listening and joint participation, functioning as a psychological buffer against anxiety and feelings of failure.

**Table 3:** Steps for Coding and Theorization (SCAT) qualitative findings matrix across the three cases. Note: PD: Parkinson's disease; GDS-15-J: Japanese version of the 15-item Geriatric Depression Scale. Step 1: Focused words/key phrases extracted from interviews; Step 2: Words outside the text for conceptualization; Step 3: Conceptual descriptions explaining the underlying mechanisms; Step 4: Major categories representing core themes. Representative verbatim quotes shown in the table are illustrative excerpts selected to demonstrate the analytical concepts and do not encompass the entirety of the raw interview transcripts. All qualitative data were originally collected and analyzed in Japanese, and representative quotes were translated into English for reporting.

Case	Major category (Step 4)	Conceptual description (Step 3)	Verbatim quotes (Step 1-2)
Case 1 Adaptation Model	1. Cognitive Restructuring and Re-meaning	Shifting subjective criteria from achievement and perfection toward functional maintenance and social meaning (e.g., skill practice, contributing to others).	"Rather than focusing on completing coloring neatly or playing saxophone tunes perfectly, I came to realize that treating them as foundational exercises for writing or muscle training for abdominal breathing was far more meaningful for me."
	2. Self-Determined Engagement	Fulfilling psychological needs (autonomy, competence, relatedness) by expanding reliance beyond family to care providers, and transforming one's identity into a supportive contributor who helps others understand PD.	"Table tennis started as balance practice, but it helped me make friends outside. I learned to rely on helper services rather than just my husband. Hearing that my helper's family member had PD, I felt that interacting with me could help them learn about PD support—realizing I could actually be helpful to others."
	3. Spousal Buffer Effect	Spouse acts as a psychological buffer against anxiety and feelings of failure by listening, sharing activities, and respecting the patient's autonomy.	"My husband always listens quietly and thinks through things with me, which is why I can keep going without giving up."
Case 2 Coexistence Model	1. Identity Continuity Through Social Recognition	Social validation from peers secures one's identity as a performing dancer, preventing role loss and psychological collapse.	"I had initially decided to quit dancing due to concerns about my physical condition and fall risks. However, when my dance peers told me, 'We want you on stage with us even in a wheelchair,' I came to realize that I still had a meaningful role within the group."
	2. Social Support Synergy	Combination of logistical support (transportation by spouse) and psychological accommodation (peer inclusion) sustains participation.	"I can continue because my husband drives me every time and my peers assist me on and off the stage."
	3. Reciprocal Transformation	Shifting mindset from "being a burden" to "coexisting," establishing a sustainable form of engagement through accepting support.	"At first, I felt distressed because I was fixated on standing and dancing alone. However, by accepting support from those around me, I was able to discover a new way of expressing myself by performing in a wheelchair."
Case 3 Conflict Model	1. Familial Risk-Avoidance Barrier and Occupational Deprivation	Overprotective family restriction acting as an environmental barrier that detaches the patient from meaningful occupations	"My family tells me to stay quiet because falling would be terrible. Even when enjoying my favorite handicraft, they stop me if my posture worsens or tremors become noticeable, which gradually made my mood darker. I just wish they would understand my illness and my feelings."
	2. Defensive Disconnection and Emotional Friction	Withdraws defensively to avoid becoming a burden; attempts home decluttering ("end-of-life prep") stall due to emotional attachment, triggering anxiety and depression.	"I started organizing things myself so as not to burden my family, but I can't throw away memories... it's deeply painful."
	3. Role Loss and Fixation as Passive Care Recipient	Loss of functional roles without alternative engagement traps the patient in a passive "care recipient" identity, fueling severe depressive symptoms (GDS-15: 10).	"My body can still move, but even things I can do are stopped... having nothing to do every day is the hardest part."

Case 2: Coexistence model through relational identity preservation

Case 2 was a woman in her early 70s, 8 years post-diagnosis, who presented with preserved cognitive function (MoCA-J: 27/30) and no depressive symptoms (GDS-15-J: 2/15). Her primary identity was deeply centered on traditional Japanese dance-a practice she had continued for 20 years, which also involved community crime prevention activities. As physical decline and fall risks progressed, she initially decided to quit dancing to avoid becoming a "burden" (meiwaku) to her group. However, her dance peers reassured her of her continued presence by proposing "wheelchair-based participation," while her husband provided ongoing transportation support to the practice sessions. Accepting this support, she shifted her mindset from insisting on standing alone to discovering a new way of expressing herself in a wheelchair in harmony with her peers.

Reflecting on this emotional journey, she stated: "I had initially decided to quit dancing due to concerns about my physical condition and fall risks. However, when my dance peers told me, 'We want you on stage with us even in a wheelchair,' I came to realize that I still had a meaningful role within the group," and "At first, I felt distressed because I was fixated on standing and dancing alone. However, by accepting support from those around me, I was able to discover a new way of expressing myself by performing in a wheelchair."

Steps for Coding and Theorization (SCAT) Analysis Results

This case illustrates a "coexistence model" for preserving occupational identity. SCAT analysis identified three major categories (Table 3):

1. Identity Continuity through Social Recognition: Peer validation and guaranteed presence as a "dancer" secured her social role, preventing identity collapse and role loss.

2. Social Support Synergy: The combination of physical infrastructure (husband's transportation) and reasonable accommodation/belonging (peer inclusion) enabled sustained engagement.

3. Reciprocal Transformation: Shifting her mindset from "being a burden" to "coexisting with supporters," establishing a new, sustainable form of performance through accepting help.

Case 3: Conflict model due to asymmetric familial risk-avoidance

Case 3 was a woman in her early 70s, 12 years post-diagnosis, who presented with severe depressive symptoms (GDS-15-J: 10/15, indicating depression) despite preserved cognitive function (MoCA-J: 29/30). Handicrafts served as a deeply meaningful occupation that allowed her to detach from her disease and express her identity. However, her husband and family viewed these activities as "unnecessary" or "fall risks," thereby restricting her engagement. Even during home handicraft activities, her family stopped her whenever her posture deteriorated or hand tremors became noticeable. To prevent familial conflict, she defensively restricted her activities and attempted home decluttering ("end-of-life planning"), but felt deeply distressed as emotional attachments to her belongings hindered progress.

Expressing her frustration and emotional isolation, she stated: "My family tells me to stay quiet because falling would be terrible. Even when enjoying my favorite handicraft, they stop me if my posture worsens or tremors become noticeable, which gradually made my mood darker. I just wish they would understand my illness and my feelings," and "My body can still move, but even things I can do are stopped... having nothing to do every day is the hardest part."

Steps for Coding and Theorization (SCAT) Analysis Results

This case illustrates a "conflict model" where family dynamics become an obstacle. SCAT analysis revealed three major categories (Table 3):

1. Familial Risk-Avoidance Barrier: Overprotective restrictions driven by risk avoidance--derived directly from quotes regarding family interrupting her handicraft during tremor episodes--acting as a major environmental obstacle.

2. Defensive Disconnection and Emotional Friction: Withdrawing defensively to avoid becoming a family burden, while failed attempts at decluttering trigger emotional friction and anxiety.

3. Role Loss and Fixation as Passive Care Recipient: Loss of functional roles without alternative engagement traps the patient in a passive "care recipient" identity, fueling severe depressive symptoms.

**Figure 1:**
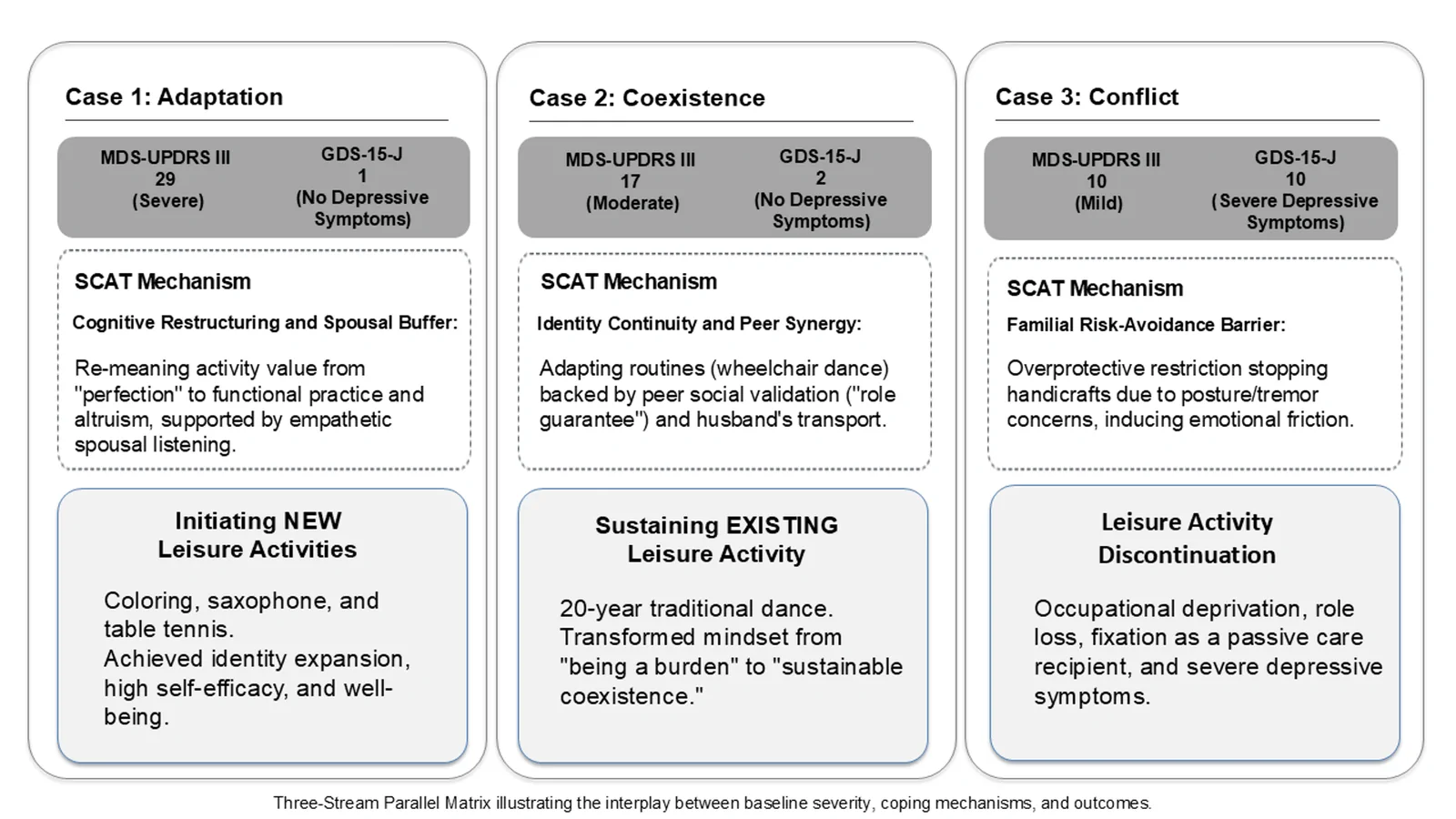
Conceptual framework of leisure engagement. Note: Conceptual framework illustrating the dynamic interplay among motor severity, coping mechanisms, and occupational outcomes in home-care patients with Stage III PD. Connecting blocks and vertical layers represent qualitative interrelationships identified through SCAT analysis rather than a deterministic temporal sequence or prospective timeline. Image Credit: Sekimoto. SCAT: Steps for Coding and Theorization; PD: Parkinson's disease; MDS-UPDRS: Movement Disorder Society-sponsored revision of the Unified Parkinson's Disease Rating Scale; GDS-15-J: Japanese version of the 15-item Geriatric Depression Scale.

A graphical summary of all three cases, mapped as a parallel conceptual framework, is presented in Figure 1.

## Discussion

The objective of this report was to structure the meanings and factors influencing the continuation or discontinuation of leisure activities among home-care patients with PD. A notable discrepancy was observed between MDS-UPDRS Part III (motor symptoms) and GDS-15-J (depressive symptoms). Paradoxically, Case 1, despite having the most severe motor symptoms, engaged in diverse activities, whereas Case 3, with the mildest symptoms, discontinued them. This finding underscores that activity persistence is not necessarily determined by physical function alone [4,14].

In progressive diseases such as PD, rigid adherence to premorbid methods often results in reduced self-efficacy due to repeated experiences of failure. In contrast, Cases 1 and 2 achieved psychological adaptation through distinct occupational pathways: Case 1 "redefined" new activity goals toward functional maintenance and social contribution, whereas Case 2 adapted her 20-year dance routine toward wheelchair-based participation to preserve identity continuity. Flexibly adjusting the value and meaning of activities, whether initiating new ones or adapting existing routines, is crucial for maintaining psychological well-being as it enables patients to experience positive personal growth and emotional fulfillment despite physical decline [7,15,16]. This observation is consistent with the selective optimization with compensation theory, which functions as a robust strategy to prevent physical decline from leading to identity loss [9].

A key finding of this study is that family and peer involvement may function either as a "buffer" or a "barrier" during progressive functional decline. As identified through the SCAT analysis (Table 3), spousal empathetic listening in Case 1 functioned as a psychological "buffer" that absorbed feelings of failure, whereas overprotective family restrictions in Case 3 functioned as an environmental "barrier" that induced occupational deprivation. This "protective instinct" inadvertently contributed to occupational deprivation [17]. A paradox emerged in which relatively better physical function was associated with accelerated role loss and activity restriction due to excessive familial protection, resulting in the patient being positioned as a passive "care recipient."

This trajectory was directly manifested in her narrative, where well-meaning family restrictions during handicraft activities ("they stop me if my posture worsens or tremors become noticeable") and the resulting loss of daily roles ("having nothing to do every day is the hardest part") led directly to profound psychological distress and severe depressive symptoms.

The stark contrast among our three cases provides a clear empirical rationale for these clinical recommendations. Cases 1 and 2 demonstrated that when patients and supporters collaboratively reconstruct the meaning of an activity-whether by initiating new pursuits or adapting 20-year routines-psychological well-being can be sustained despite severe motor impairment. Conversely, Case 3 illustrated that individual motivation is easily overwhelmed if family members enforce complete risk elimination, leading directly to occupational deprivation and severe depressive symptoms. Therefore, interventions targeting "activity" and "participation" must extend beyond physical training to incorporate value reconstruction for both patients and families. The multidisciplinary home-care team-including physicians, nurses, physical therapists, occupational therapists, and care managers-should emphasize the meaning of life beyond the activity itself and support value transitions in accordance with functional changes. At the same time, it is essential for the team to educate family members that well-intentioned support may unintentionally restrict patient autonomy. Sustaining leisure activities requires shared decision-making that acknowledges rather than eliminates risk, thereby facilitating self-management through a shared recognition of activity significance [18-20].

Study limitations

This study has several limitations that should be acknowledged. First, because this report is based on a small, qualitative case series of three home-care patients with PD, the findings are exploratory in nature and cannot be widely generalized to the broader population with PD. Second, the use of purposive sampling (maximum variation) may have introduced selection bias, as cases were specifically chosen to illustrate contrasting patterns of leisure engagement. Third, qualitative data analysis via SCAT inherently involves subjective interpretations by the researchers, which may influence the framing of categories and models. Finally, as a cross-sectional study, chronological descriptions of past leisure activity changes rely on participants' retrospective accounts during interviews rather than prospective longitudinal monitoring. Future large-scale longitudinal studies incorporating diverse clinical stages and family environments are warranted to validate these qualitative findings.

## Conclusions

In conclusion, this qualitative case series suggests that leisure activities among home-care patients with PD can be transformed into a sustainable form of coexistence, regardless of physical severity, provided that there is an appropriate "redefinition of meaning" and the establishment of shared values with supporters. Based on observations from these three cases, psychological well-being in progressive illness appears to rely not solely on residual physical capacity but also significantly on the subjective reconstruction of meaning and on collaborative family support. In multidisciplinary home care, addressing these relational and psychological dimensions across healthcare professions may be valuable for enhancing overall quality of life.

## References

[1] Chaudhuri KR, Healy DG, Schapira AH (2006). Non-motor symptoms of Parkinson's disease: diagnosis and management. Lancet Neurol.

[2] Zhao N, Yang Y, Zhang L (2021). Quality of life in Parkinson's disease: a systematic review and meta-analysis of comparative studies. CNS Neurosci Ther.

[3] Cassidy I, Doody O, Richardson M, Meskell P (2024). Quality of life and living with Parkinson's disease: a qualitative exploration within an Irish context. BMC Neurol.

[4] Sjödahl Hammarlund C, Westergren A, Åström I, Edberg AK, Hagell P (2018). The impact of living with Parkinson's disease: balancing within a web of needs and demands. Parkinson's Dis.

[5] Sekimoto M, Takeshima R, Kadota A (2025). Relationship between the duration of leisure activities at home and nonmotor symptoms among people with Parkinson's disease. Asian J Hum Serv.

[6] Goetz CG, Poewe W, Rascol O (2004). Movement Disorder Society Task Force report on the Hoehn and Yahr staging scale: status and recommendations. Mov Disord.

[7] Wieringa G, Dale M, Eccles FJ (2022). Adjusting to living with Parkinson's disease; a meta-ethnography of qualitative research. Disabil Rehabil.

[8] Chiong-Rivero H, Ryan GW, Flippen C (2011). Patients' and caregivers' experiences of the impact of Parkinson's disease on health status. Patient Relat Outcome Meas.

[9] Baltes PB, Baltes MM (1990). Psychological perspectives on successful aging: the model of selective optimization with compensation. Successful Aging: Perspectives from the Behavioral Sciences.

[10] Otani T (2019). Principles of Qualitative Data Analysis: From Research Methodology to Steps-for-Coding-and-Theorization (SCAT).

[11] Goetz CG, Tilley BC, Shaftman SR (2008). Movement Disorder Society-sponsored revision of the Unified Parkinson's Disease Rating Scale (MDS-UPDRS): scale presentation and clinimetric testing results. Mov Disord.

[12] Sugishita K, Sugishita M, Hemmi I, Asada T, Tanigawa T (2017). A validity and reliability study of the Japanese version of the Geriatric Depression Scale 15 (GDS-15-J). Clin Gerontol.

[13] Fujiwara Y, Suzuki H, Yasunaga M (2010). Brief screening tool for mild cognitive impairment in older Japanese: validation of the Japanese version of the Montreal Cognitive Assessment. Geriatr Gerontol Int.

[14] Menon B, Nayar R, Kumar S, Cherkil S, Venkatachalam A, Surendran K, Deepak KS (2015). Parkinson's disease, depression, and quality-of-life. Indian J Psychol Med.

[15] Mathur S, Mathur S (2024). Patient empowerment for those living with Parkinson's disease. J Parkinson's Dis.

[16] Tuijt R, Tan A, Armstrong M (2020). Self‐management components as experienced by people with Parkinson's disease and their carers: a systematic review and synthesis of the qualitative literature. Parkinson's Dis.

[17] Whitehead L, Jacob E, Towell A, Abu-Qamar M, Cole-Heath A (2018). The role of the family in supporting the self-management of chronic conditions: a qualitative systematic review. J Clin Nurs.

[18] McCarron TL, Noseworthy T, Moffat K (2020). A co-designed framework to support and sustain patient and family engagement in health-care decision making. Health Expect.

[19] Jones J, Ramaswamy B (2025). Empowering people with Parkinson's: reframing self-management in Parkinson's-a critical reflection of current practice. Healthcare (Basel).

[20] Milne-Ives M, Carroll C, Meinert E (2022). Self-management interventions for people with Parkinson disease: scoping review. J Med Internet Res.

